# Neutrophil extracellular trap-enriched supernatants carry microRNAs able to modulate TNF-α production by macrophages

**DOI:** 10.1038/s41598-020-59486-2

**Published:** 2020-02-17

**Authors:** Leandra Linhares-Lacerda, Jairo Ramos Temerozo, Marcelo Ribeiro-Alves, Estefania P. Azevedo, Andres Mojoli, Michelle T. C. Nascimento, Gustavo Silva-Oliveira, Wilson Savino, Debora Foguel, Dumith Chequer Bou-Habib, Elvira M. Saraiva

**Affiliations:** 1 Laboratory of Immunobiology of Leishmaniasis, Department of Immunology, Instituto de Microbiologia Paulo de Góes, Universidade Federal do Rio de Janeiro, Rio de Janeiro, Brazil; 2 Laboratory on Thymus Research, Oswaldo Cruz Institute, Oswaldo Cruz Foundation, Rio de Janeiro, Brazil; 3 National Institute of Science and Technology on Neuroimmunomodulation, Oswaldo Cruz Institute, Oswaldo Cruz Foundation, Rio de Janeiro, Brazil; 4 HIV/AIDS Clinical Research Center, Evandro Chagas National Institute of Infectology, Oswaldo Cruz Foundation, Rio de Janeiro, Brazil; 5 Instituto de Bioquimica Médica Leopoldo de Meis, Universidade Federal do Rio de Janeiro, Rio de Janeiro, Brazil Brazil

**Keywords:** Cellular imaging, Granulocytes

## Abstract

Neutrophil extracellular traps (NETs) emerge from the cell as a DNA scaffold associated with cytoplasmic and granular proteins, able to immobilize and kill pathogens. This association occurs following nuclear and granular membrane disintegration, allowing contact with the decondensed chromatin. Thus, it is reasonable to speculate that the DNA can also mix with miRNAs and carry them in NETs. Here, we report for the first time the presence of the miRNA carriers associated with NETs and miRNAs present in NET-enriched supernatants (NET-miRs), thus adding a novel class of molecules and new proteins that can be released and transported in the NET platform. We observed that the majority of NET-miRs were common to all four stimuli used (PMA, interleukin-8, amyloid fibrils and *Leishmania*), and that miRNA-142-3p carried by NETs down-modulates protein kinase Cα and regulates TNF-α production in macrophages upon NET interaction with these cells. Our findings unveil a novel role for NETs in the cell communication processes, allowing the conveyance of miRNA from neutrophils to neighboring cells.

## Introduction

Neutrophils are pivotal cells of the innate immune response, as they are the first leukocytes to reach infected or injured tissues. Neutrophils are endowed with powerful microbicidal properties, such as phagocytosis, degranulation and extrusion of neutrophil extracellular traps (NETs)^1^. NETs are scaffold of chromatin decorated with cytoplasmic and granular proteins released to the extracellular milieu that are able to immobilize and kill pathogens by means of toxic molecules such as elastase and histone^2^. NET release is triggered by different stimuli, such as pathogens (bacteria, fungi, viruses, parasites)^2^, endogenous molecules (e.g., interleukin [IL]-8 and amyloid fibrils)^2,3^, and inorganic compounds, such as phorbol myristate acetate (PMA)^1^.

The NET extrusion process is initiated with the loss of the classical nuclear morphology and chromatin decondensation, followed by the disappearance of all internal membranes, allowing the assembly of NET components^4^. Many granular and cytoplasmatic proteins have been identified as NET cargos^5^, but the complete NET components remain to be defined. Thus, we asked whether other molecules, such as microRNAs (miRNAs), could be associated with NET scaffolds. miRNAs are short (19–24 nucleotides in length) non-coding RNAs, found intracellularly and outside the cells, and that regulate messenger RNA (mRNA) or protein levels either by promoting mRNA degradation or by attenuating protein translation. Despite accumulating evidence of extracellular miRNAs^6,7^, their presence associated with NETs has not yet been described.

## Results

### NETs carry small RNAs

**Figure 1:**
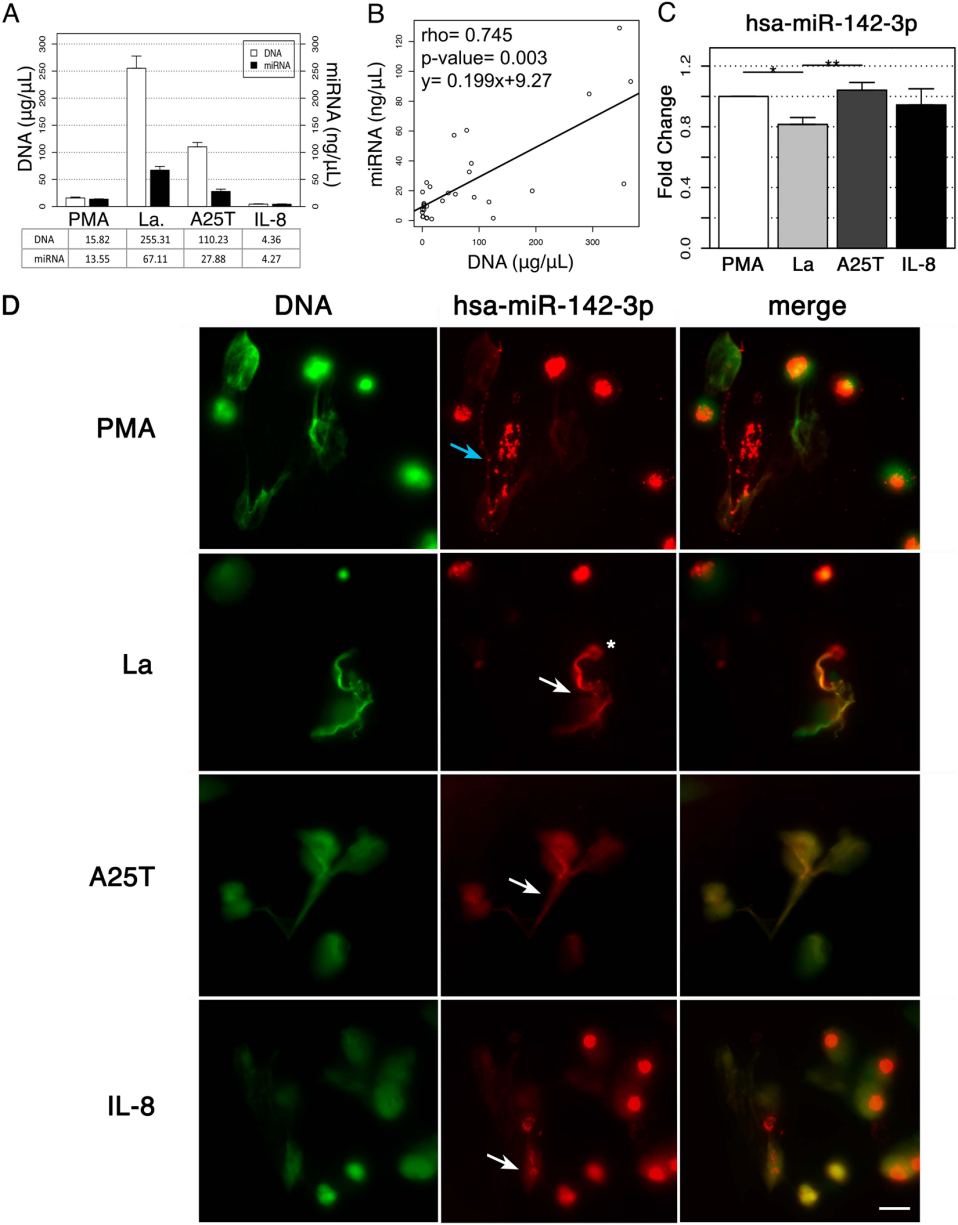
NETs carry miRNA cargo. Neutrophils from healthy donors were exposed to four different activators, including PMA (100 nM), amyloid fibrils (A25T, 3 μM), IL-8 (50 ng/mL) and *L. amazonensis* promastigotes (La; ratio of 5 parasites/neutrophil) for 3 h. (**A**) Measurements obtained from DNA (PicoGreen dsDNA kit) and miRNA (Agilent 2100 RNA Bioanalyzer) for each stimulus. (**B**) Correlation between DNA and miRNA quantities from A. (**C**) Quantitative PCR results for the hsa-miR-142-3p Taqman assay displaying the mean expression levels ± SEM of the test groups (La and A25T or IL-8) in relation to the control (PMA) in donor-paired analysis from 6 independent experiments for each activator. Statistical significance was calculated by a two-tailed test (**p < 0.001; *p < 0.05). **(D)** Live-cell imaging of NET formation in two different channels for up to 120 minutes. SYTOX Green at 10 nM was used for NET detection (depicted in green; left panels), and a TYE™-665 labeled hsa-miR142-3p locked nucleic acid (LNA) detection probe at 5 μM for miR-142-3p detection (depicted in red; middle panels). The hsa-miR-142-3p staining was present in two different morphological patterns: one shows a strong staining (asterisk), and the other reveals a weak staining that can be either punctate (blue arrow) or diffuse (white arrows). The right panels show the merge of the two channels. Bars: 20 μm.

During NET formation, the decondensed chromatin comes into contact with cytoplasmic and granular components before cell membrane rupture^4^, making it plausible to hypothesize that NETs can carry neutrophil intracellular miRNAs. Therefore, we first evaluated whether miRNAs could be associated with NETs. Human neutrophils from healthy donors were stimulated with four previously reported NET inducers including the chemical agent phorbol myristate acetate (PMA), amyloid fibrils composed of transthyretin variant (A25T), the inflammatory mediator Interleukin (IL)-8, and the pathogen parasite *Leishmania amazonensis*. Following neutrophil activation, the DNA and small RNA contents of NET-enriched supernatants were quantified, and we detected that NET-containing supernatants from all stimuli carried substantial quantities of small RNAs, including those of 19 to 24 nucleotides in length, which have been classified as miRNAs (Supplementary Fig. S1). The amounts of DNA and miRNA in NET-enriched supernatants strongly correlated (Fig. 1A,B), suggesting that the DNA quantity can provide an indirect estimation of the miRNA cargo in NETs. To assure that the DNA and small RNA contents derived from NETotic cells, thus ruling out a possible contamination from non-NETotic lytic cells, we evaluated the presence of the cell rupture marker lactate dehydrogenase (LDL) in NET preparations. We found that LDL release was not observed after three hours of neutrophil activation and NET formation (Supplementary Fig. S2), similar to what has been described by other authors^1,8–10^.

We next searched the NET components for the presence of a particular miRNA. Because the expression of miR-142 has been described in myeloid lineages and particularly in neutrophils, where it plays a role in cell maturation^11^, the miR-142 was chosen as the first candidate for further analysis. To directly determine whether miR-142-3p (the functional form of miR-142) was present in NETs, NET-enriched supernatants were used as input samples, and a Taqman quantitative RT-PCR assay was performed. These approaches allowed the detection of the miR-142-3p, thus confirming that mature miRNAs were present in the NETs induced by all neutrophil activators used here (Fig. 1C). Interestingly, the amounts of miR-142-3p present in PMA- and A25T-induced NETs were significantly higher than those found in NETs induced by *Leishmania*. In addition, NET formation was directly monitored by live-cell imaging in the presence of SYTOX Green (to visualize NETs) and miR-142-3p-probe. As shown in Fig. 1D, NETs were detected after neutrophil activation by all stimuli (green, NET-DNA staining). Moreover, miR-142-3p appeared as a strong red staining that colocalized with NETs (Fig. 1D merged images) with two different morphological patterns, either as punctate staining associated with the DNA fibers in PMA-induced NETs (Fig. 1D, blue arrow), or as a diffuse pattern with the three other NET activators (Fig. 1D, white arrows). Taken together, these results clearly demonstrate that NETs carry miR-142-3p.

### hsa-miR-142-3p in NET formation steps

Previous analysis of the DNA architecture defined four distinct morphologies that have been observed during the progression of NET formation, such as lobulated and delobulated nuclei, diffused and spread NET^12^.

**Figure 2:**
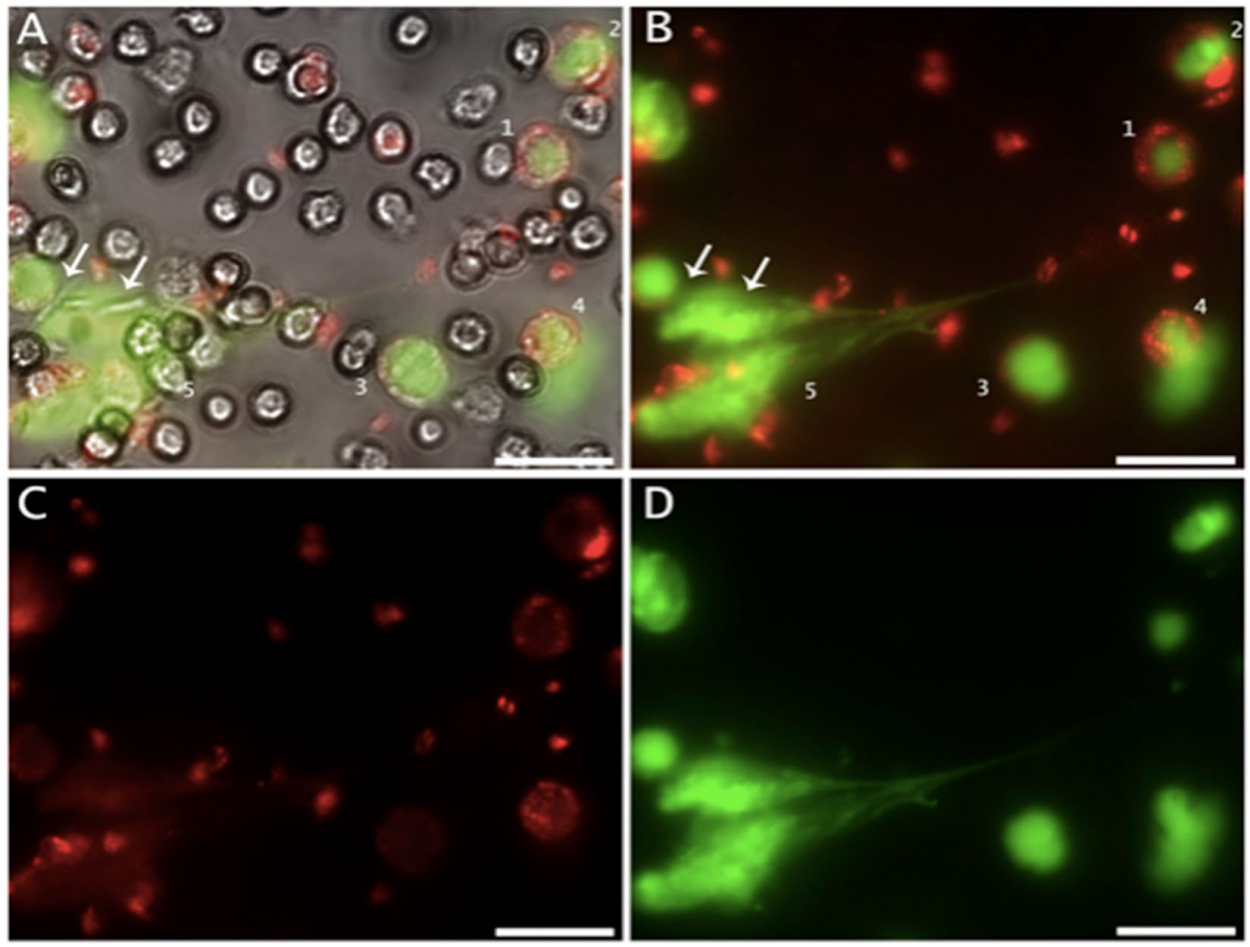
miR-142-3p staining pattern throughout NET formation steps. Neutrophils were activated with fixed *L. amazonensis* promastigotes (La; ratio of 5 parasites/neutrophil) for up to 120 min, and directly monitored *in vitro* by live-cell imaging using differential interference contrast (DIC), SYTOX Green as a NET marker and LNA-miR-142-3p-TYE™665 as a miRNA marker. **(A,B)** The morphological characteristics of NET formation are represented, depicting the loss of the classical neutrophil lobulated nuclei with decondensed chromatin, as well as hsa-miR-142-3p staining (red) as a diffuse, punctate pattern, in the neutrophil cytoplasm (1). A cell with the nuclear membrane starting to disintegrate (2) and another with amorphous nuclear material filling most of the cell (3) can be seen. The images also show the extrusion of a diffuse NET (4), and a spread-out NET with the DNA scaffold forming a web-like structure, with hsa-miR-142-3p staining (red) colocalized with DNA (green) (5). Two NET-trapped *Leishmania* promastigotes (Fig. A, arrows) and non-stained viable neutrophils were observed. (**C**) LNA-miR-142-3p-TYE™665 (5 μM) detection (red). (**D**) NET staining by SYTOX Green (10 nM). Bars: 25 μm.

**Figure 3:**
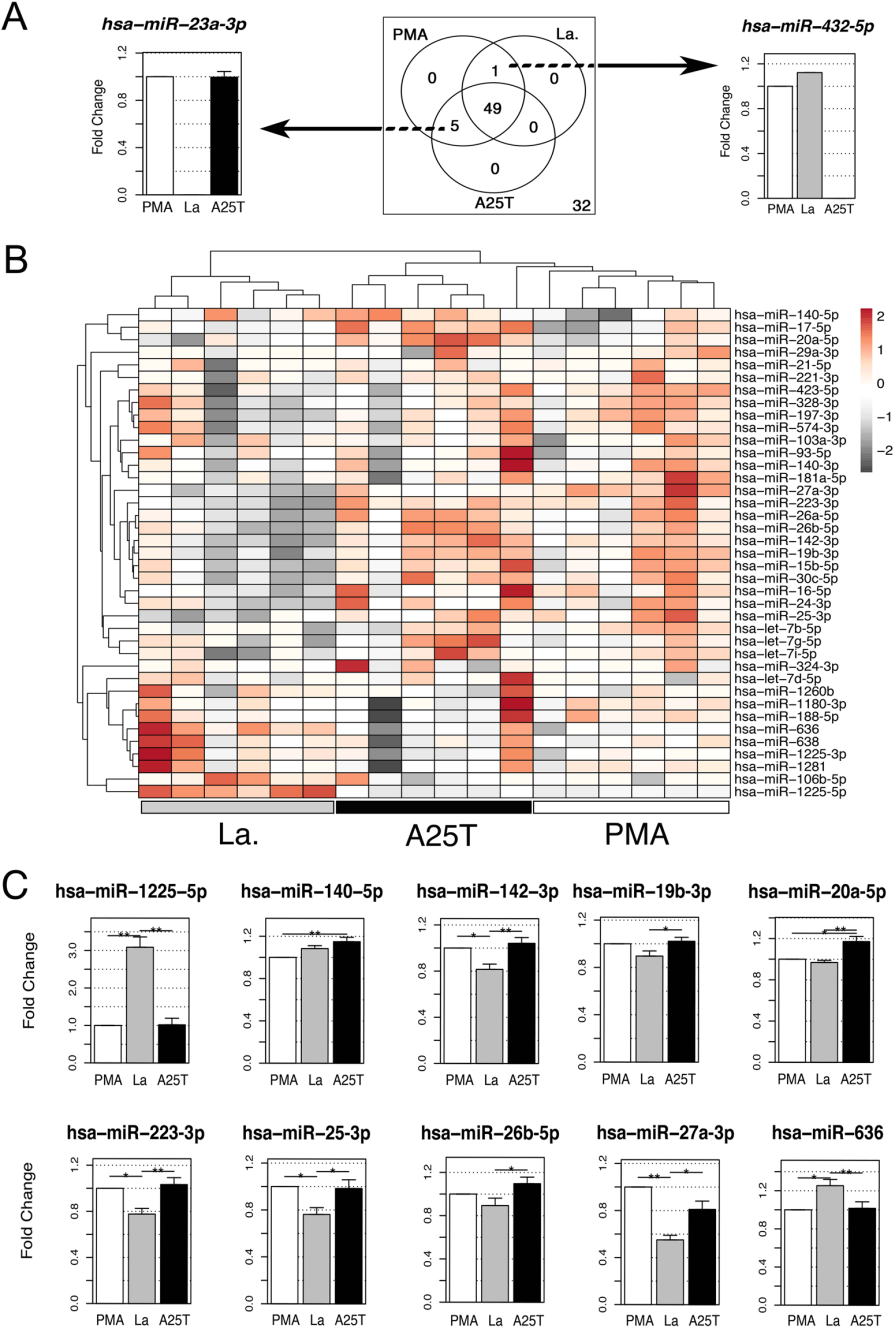
NET-associated miRNAs (NET-miRs) present differential patterns upon distinct types of stimuli. We constructed a miRNA expression profile for 87 different Taqman assays obtained with quantitative PCR from NET supernatants obtained with three activators (PMA, A25T, La). (**A**) Venn diagram (center) showing the number of amplified miRNAs for each stimulus; the number zero corresponds to non-detected miRNAs. The left graphic depicts an example of a miRNA that was amplified with PMA and A25T treatment, and the right graphic shows hsa-miR-432-5p, which was amplified only in the PMA and La samples. (**B**) Profiling of the expression values of 39 miRNAs from *Leishmania* (La, gray box), amyloid fibrils (A25T, black box) and PMA (white box) stimulated neutrophil supernatants. The color scale shown on the right illustrates the expression from the normalized data, in which red indicates an expression level higher than the mean across all subjects, gray denotes an expression level lower than the mean, and white indicates the median expression. Hierarchical cluster analysis subdivided the samples into three main groups, in which A25T and PMA treatments are clustered together in the dendrogram at the top of the heatmap, while the dendrogram on the left illustrates the miRNA clustering. (**C**) Bar plots showing the miRNA expression difference between the activators, displaying the mean expression levels ± SEM of the test groups (La and A25T) relative to the control group (PMA). To avoid donor-to-donor variation, we performed donor-paired analysis for the three NET activators. Statistical significance was calculated by comparison of the means of the normalized gene expression values between groups performed by a nonparametric one-way ANOVA with 1,000 unrestricted permutations, followed by post-hoc pair-wise comparisons with Bonferroni adjustment using a nonparametric t-test also with 1,000 permutations (**p < 0.001) and (*p < 0.05). Results are from 6 independent biological replicates for each activator. PMA, phorbol myristate acetate; A25T, amyloid fibrils; La, *L. amazonensis* promastigotes.

To understand the miR-142-3p dynamics during NET formation, *Leishmania-*stimulated neutrophils were directly monitored by live-cell imaging, using SYTOX green as a NET marker and a miR-142-3p-probe. This approach allowed us to identify this miRNA during the typical NET formation steps. The delobulated nuclei step is depicted in Fig. 2 (numbers 1, 2 and 3), in which the DNA staining shows a representative morphology of decondensed chromatin, and the miR-142-3p staining appears diffuse, with a punctate pattern in the neutrophil cytoplasm. The picture also illustrates a diffused NET morphology (Fig. 2, number 4), showing the released chromatin. Lastly, a spreading NET morphology with a DNA scaffold forming a classical web-like structure is shown (Fig. 2, number 5). Notably, a miR-142-3p staining can colocalize with the DNA (green) (Fig. 2C,D), as well as three NET-trapped *Leishmania* promastigotes (Fig. 2A, arrows) and non-stained viable neutrophils (Fig. 2A) are perceived. In order to exclude unspecific binding of the probe due to the adhesive nature of NET, we labeled *Leishmania-*induced NETs with a probe specific for miR-181a-5p, which is poorly present in these NETs (Fig. 3B). Our results revealed NETs with the DNA scaffold forming a web-like structure containing trapped *Leishmania* promastigotes without miR-181a-5p specific staining, which was observed only in artifacts/dead neutrophils (Supplementary. Fig. S3).

The cytoplasmic miR-142-3p localization pattern during NET formation here described may represent only the content of cytosolic miRNAs. Because miRNA subcellular localization is dynamic and the miRNAs are partitioned into different intracellular pools^13^, we cannot rule out the hypothesis that NETs can carry miRNAs from other cellular locations, such as cytoplasmic organelles, nucleus and nucleolus.

### Differential patterns in NET-associated miRNAs in response to distinct types of stimuli

The finding that NETs can carry miR-142-3p prompted us to search for other NET-associated miRNAs. Thus, to select putative candidates, we screened for miRNAs that were significantly expressed in activated neutrophils, irrespective of the activator (Supplementary Table S1), using reanalysis of two microarray data sets available in the databases E-TABM-1097^14^ and GSE18999^15^. From those data sets, we selected 87 miRNAs to search for in NETs using a quantitative PCR array. To avoid donor-to-donor variation, we performed donor-paired analysis for three NET activators previously described, using PMA as a standard. Because IL-8-induced NETs had a small number of miRNAs (Fig. 1A), they were excluded from the PCR array assays; nonetheless, the miRNAs let-7b-5p, miR-17-5p and miR-26a-5p were detected in these samples (Supplementary Fig. S4), demonstrating that IL-8 also induces miRNA release associated with NETs. Regardless of the type of PCR used (array or not), all samples were mixed with three different synthetic *C. elegans* miRNAs to allow normalization of sample-to-sample variation, a methodology already used for miRNA screening in plasma samples^6^ (see Methods section). The PCR array screen detected 55 miRNAs associated with NET-enriched supernatants, of which only one was not present in NETs triggered by amyloid fibrils, and five miRNAs were absent in the *Leishmania*-induced NETs (Fig. 3A and Supplementary Table S2). The relative quantification of the detected miRNAs is illustrated in the dendrogram (Fig. 3B). Moreover, unsupervised clustering demonstrated that each stimulus presents miRNA patterns that are distinct enough to divide the sample groups into different clusters. Indeed, the relative amounts of some of the miRNAs were significantly different between the three sample groups, most notably when we compared *Leishmania*-induced NETs with the other NET activators (Fig. 3C). This was the case for miR-1225-5p and miR-636, which were upregulated, and miR-142-3p, miR-223-3p, miR-25-3p and miR-27a-3p, which were downregulated, in NET triggered by *Leishmania* (Fig. 3C). Furthermore, in NET samples induced by amyloid fibrils, miR-140-5p and miR-20a-5p were significantly upregulated when compared to PMA, and miR-19b-3p, miR-20a-5p and miR-26b-5p were upregulated relative to the *Leishmania*-stimulated samples. Although 32 of the selected miRNAs exhibited a consistent pattern of no amplification (Supplementary Table S1), we confirmed the presence of miRNA cargo in NETs, hereafter designated NET-associated miRNAs or NET-miRs.

### Extracellular miRNA carriers are present in NETs

**Figure 4:**
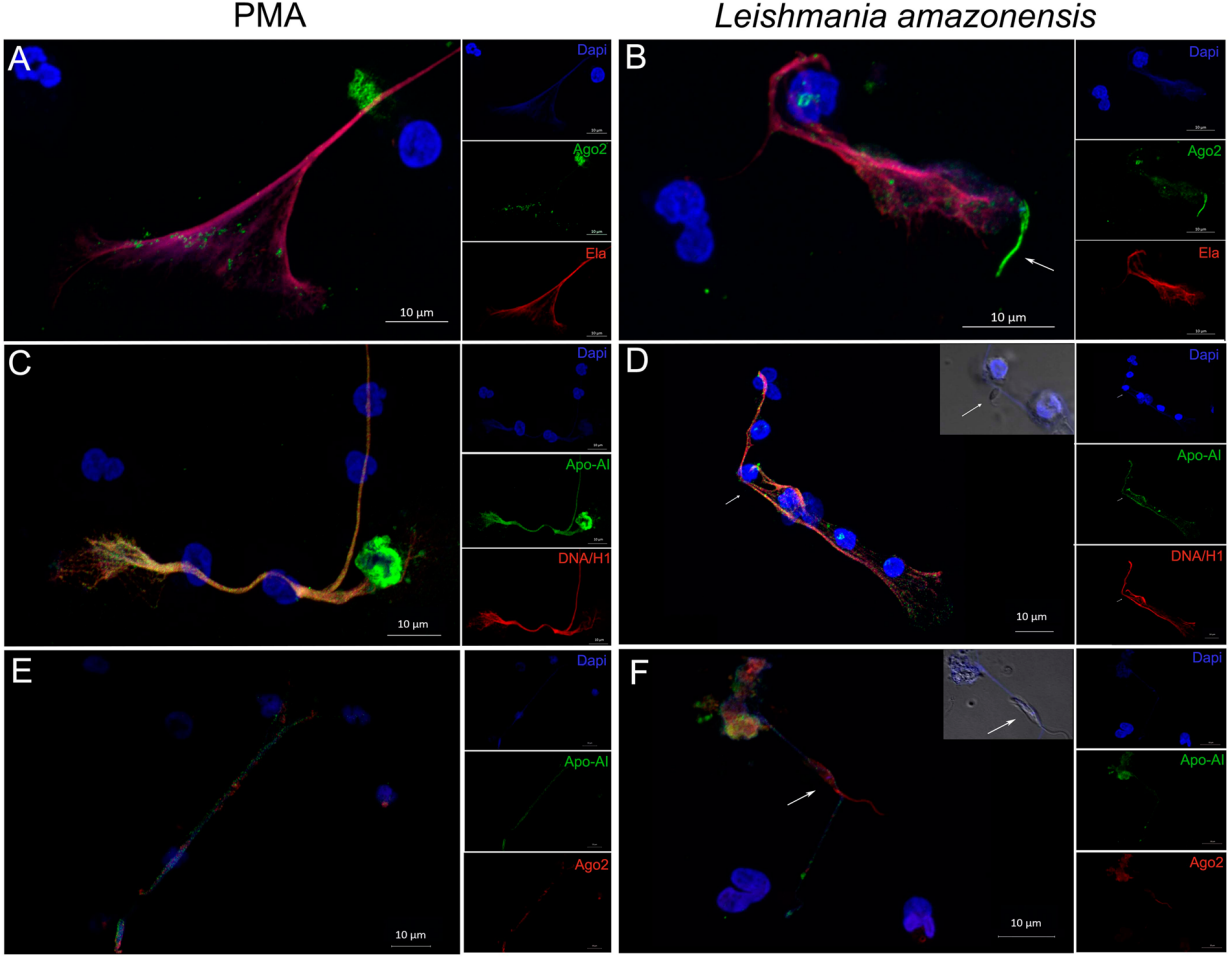
Extracellular miRNA carriers are present in NETs. NET induced by PMA (**A**,**C**,**E**) or fixed *L. amazonensis* promastigotes (La; ratio of 5 parasites/neutrophil) (**B**,**D** and **F**) were immunostained with Argonaute-2 (Ago-2), Apoliprotein-AI (Apo-AI), Elastase (Ela), DNA/Histone H1 (DNA/H) and DAPI followed by Alexa fluor 488 or 546 secondary antibodies. Dapi staining images merged with differential interference contrast are shown in *Leishmania*-treated samples showing NET ensnared promastigote (inserts, arrows). Bars: 10 μm.

Extracellular miRNAs can be found in protein-bound complexes (such as Argonaute-2; Ago2) or packaged into lipid-based carriers, such as exosomes or apoptotic bodies, and also on high- and low-density lipoproteins (HDL and LDL, respectively)^16,17^. Taken these findings into account, we wondered whether some of these complexes were present in NETs. We therefore stained NETs triggered by PMA or *L. amazonensis* for DAPI, Ago2, Apoliprotein-AI (Apo-AI, which is the HDL main protein component), elastase and DNA/histone H1. We found that both Ago2 and Apo-AI colocalize with the NET components elastase (Fig. 4A,B) and DNA/histone (Fig. 4C,D), respectively. Of note, Ago2 and Apo-AI were detected in different locations of the NETs web-like structure (Fig. 4E,F).

### NET-associated miRNA-142-3p regulates PKCα expression in macrophages

**Figure 5:**
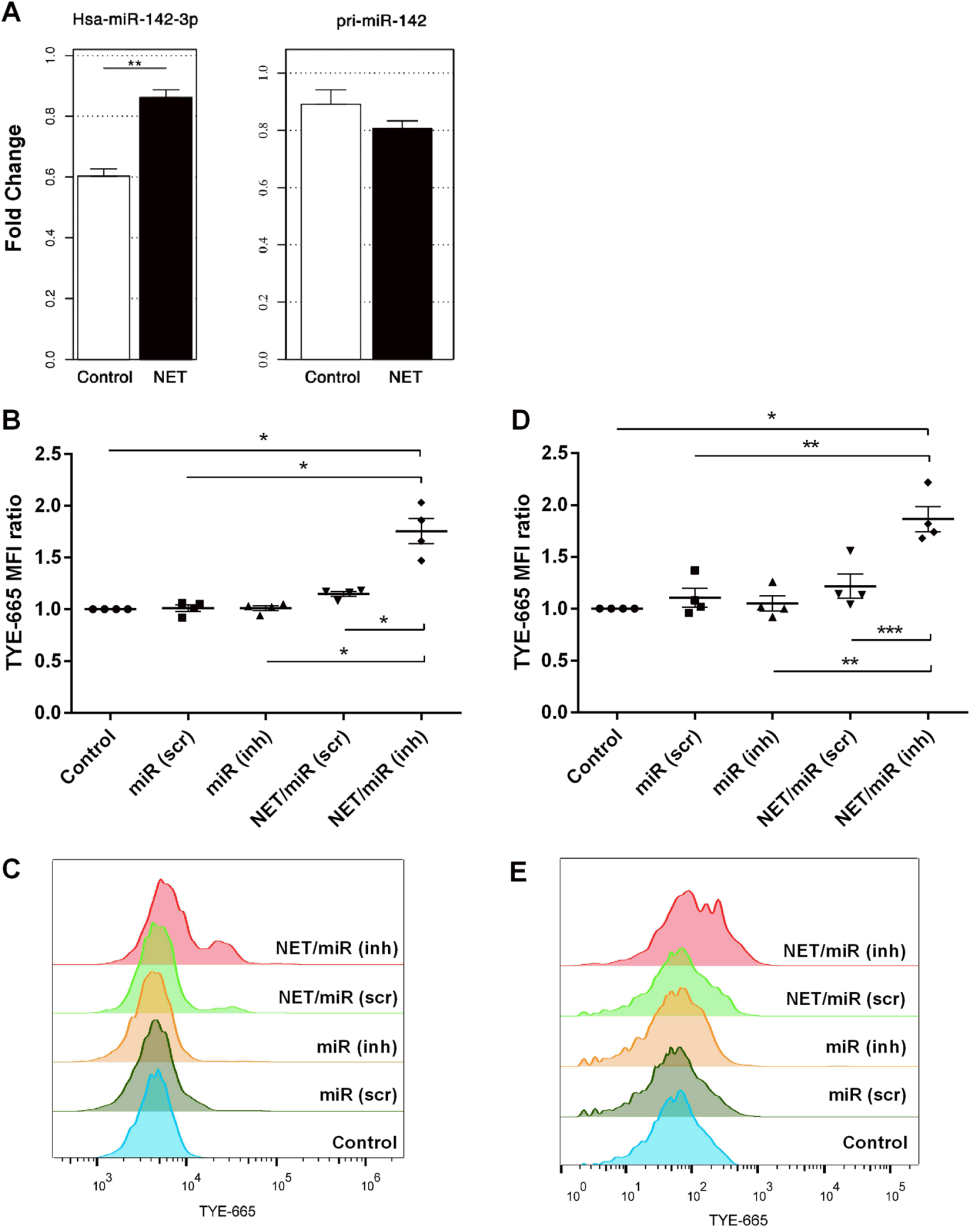
NET-mediated transfer of miRNA-142-3p to macrophages. (**A**) PMA-differentiated THP-1 macrophages were exposed for 2 h to PMA-induced NET, following washing and complete RNA extraction for hsa-miR-142-3p (left group) and hsa-pri-miR-142 (right group) expression evaluated by quantitative PCR using Taqman probes. (**B**–**E**) PMA-differentiated THP-1 macrophages were exposed for 2 h to PMA-induced NET associated with specific hsa-miRNA-142-3p inhibitor or scramble sequence control, or to the hsa-miRNA-142-3p inhibitor or the scramble sequence control alone. After, cells were washed, and complete culture medium was added. Two hours (**B**,**C**) or 18 h later (**D**,**E**), cells were analyzed through flow cytometry for detection of hsa-miRNA-142-3p inhibitor or scramble sequence control in cells; both being labeled with the TYE™-665 fluorochrome. Data are shown as ratio of mean fluorescence intensity (MFI). Results in (**A**) are from 5 independent experiments using paired Student’s *t* test, and in (**B**,**D**) are from 4 independent experiments using one-way ANOVA, with Bonferroni post-hoc test, displaying mean ± standard error of mean of the test group. *p < 0.05; **p < 0.01; ***p < 0.001; ****p < 0.0001.

**Figure 6:**
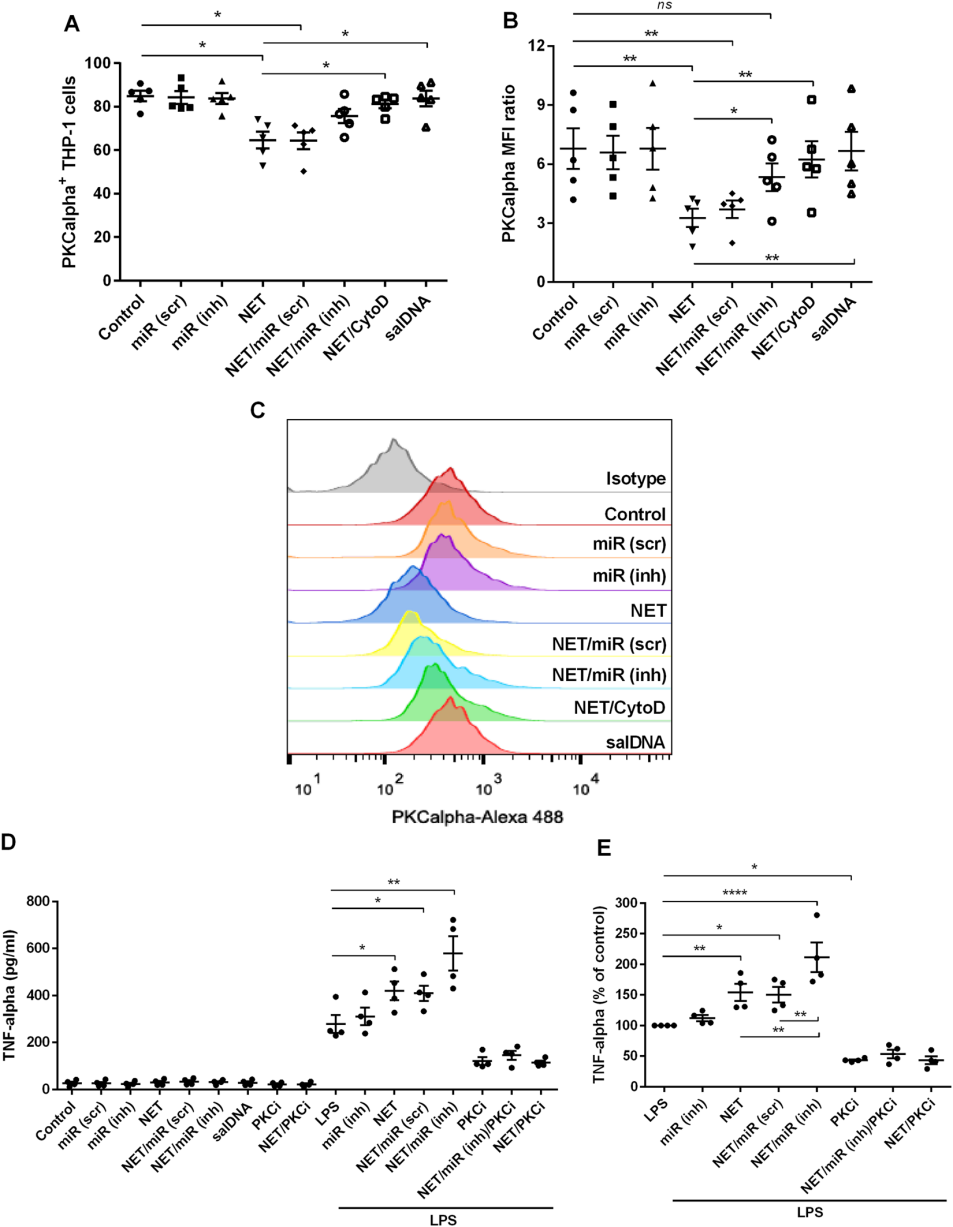
NET-associated miRNA-142-3p regulates PKCα expression in macrophages. (**A**,**B**) PMA-differentiated THP-1 macrophages were exposed to PMA-induced NET incubated or not with specific TYE-665-labelled hsa-miRNA-142-3p inhibitor or scramble sequence control, or treated with cytochalasin D (CytoD) before NET exposure. Salmon sperm DNA (salDNA) was used as control. After 2 h, cells were washed and complete culture medium was added. The expression of PKCα was evaluated using flow cytometry, showing positive population (**A**) and median fluorescence intensity ratio (MFI ratio, and **B**) 18 h after treatment, with (**C**) as a representative histogram. (**D**,**E**) THP-1 macrophages were exposed to NET associated or not with specific hsa-miRNA-142-3p inhibitor or scramble sequence control, or treated with salmon sperm DNA, or a PKC inhibitor (iPKC, Go6383, 50 nM). Cells were also treated with LPS (20 ng/ml) plus treatments above. After 2 h, cells were washed and complete culture medium was added. Culture supernatants were collected 6 h later and TNF-α production was quantified by ELISA. (**E**) Analysis of TNF-α production when normalized to levels induced by LPS. Results (**A**,**C**,**D**) derive from 4 independent experiments using one-way ANOVA, with Bonferroni post-hoc test, displaying mean ± standard error of mean of the test group. *p < 0.05; **p < 0.01; ***p < 0.001; ****p < 0.0001.

In our previous analysis, we identified several miRNAs in NETs derived from different stimuli (Fig. 3C) and found that miRNA carriers are also present in those NETs (Fig. 4). Thus, it seemed possible that, upon NET interaction with adjacent cells, those miRNAs could be transferred and then modulate the expression of their correspondent mRNA targets in recipient cells. Amongst the miRNAs identified, miRNA-142-3p has been described as a negative regulator of signaling pathways, leading to reduced levels of inflammatory cytokines in different cell types^18–21^. miRNA-142-3p down regulates the isoform α of the Protein Kinase C (PKC)^22^, which is one of the major regulators of macrophage function following activation by PAMPs, such as LPS. Several PKC isoforms, including PKCα, are engaged in the signaling pathway leading to the production of IL-6, TNF-α and other proinflammatory mediators^23–26^. Because macrophages interact with NETs, promoting their clearance and participating in the inflammatory response^27^, we investigated whether the miRNA-142-3p could modulate cellular response after macrophage interaction with NETs. Initially, we exposed the human monocytic leukemia cell line THP-1, differentiated into macrophages by PMA treatment to NET-enriched supernatants. We then evaluated NET-mediated transfer of miRNA-142-3p, by using quantitative PCR for its primary and mature sequence. As can be seen in Fig. 5A, we only observed increase in the mature form. This finding indicates that its presence was not a product of a new synthesis, but rather acquired directly in its mature form (Fig. 5A, left group). Also, through flow cytometry, we detected miRNA-142-3p in cells exposed to NET associated to specific fluorescent miRNA-142-3p inhibitor probe, in both 2 and 18 h post-incubation (Fig. 5B–E), indicating its transfer and permanency in cells, which in turn points to a possible functional activity. To deepen this issue, we exposed PMA-differentiated THP-1 macrophages to NETs, associated or not with specific miRNA-142-3p inhibitor probe or scramble sequence control and, 18 h later, we evaluated PKCα expression by flow cytometry. We detected that PKCα expression was reduced in NET-treated macrophages and that this reduction was partially reverted in the presence of the specific miRNA-142-3p inhibitor probe. In addition, the PKCα decrease did not occur when cells were treated with cytochalasin D before NET exposure, pointing that miRNA transfer was dependent of actin-mediated internalization (Fig. 6A–C).

Following, we analyzed the levels of TNF-α in supernatants from THP-1 cells exposed to NETs or to NETs plus LPS, associated or not with specific miRNA-142-3p inhibitor probe or scramble sequence control, after 6 h. Induction of TNF-α by NETs occurred only when they were associated with LPS, with NETs promoting a increment in LPS-induced-TNF-α production, similar to previous data showing that clearance of NETs is a “silent process”^28^. The production of TNF-α was exacerbated when LPS-treated cells were exposed to NETs previously incubated with specific miRNA-142-3p inhibitor probe (Fig. 6D,E). The induction of TNF-α by NETs/LPS, and the increment of TNF-α levels found when miRNA-142-3p was inhibited, were abrogated in the presence PKC inhibitor, indicating that the phenomenon observed was dependent of PKC activation by NET/LPS, and that the miRNA-142-3p can attenuate TNF-α production by down-regulating PKC. Our findings demonstrate that NETs are decorated with miRNA and miRNAs transporters, and that the miRNA-142-3p carried by NETs modulates PKCα in macrophages, thus indicating that NETs participate in cell-to-cell communication, transferring miRNAs and modulating cellular processes.

## Discussion

Until now, only the association of DNA with proteins has been found in the web-like NET structure^5^. Herein, we report the detection of miRNA carriers associated with NETs and miRNAs in NET-enriched supernatants, thus adding a novel class of molecules and new proteins that can be released by netting neutrophils and transported in the NET platform.

The secretion of miRNAs is a controlled, active and specific process; however, the mechanisms of the selectivity of miRNA packing remain unclear^29^. We found that the majority of NET-miRs were conserved regardless of the different stimuli used and, although some of them were absent following neutrophil exposure to *Leishmania*, the expression levels of miRNAs were diverse in response to each activator, suggesting that the miRNA cargo varies depending on the specific stimulus that induces NETs.

Conceptually, these findings suggest the existence of a physiological regulation of NET-based miRNA transportation to the extracellular milieu. In this context, more studies are necessary to define whether differential NET-miR release reflects the molecular changes triggered by each activator (i.e., inducing modifications in the neutrophil miRNA content) or if it is an active sorting process that takes part in NET formation. In fact, a selective release of specific endogenous miRNAs by a trafficking system has already been proposed, in which miRNAs are specifically packaged into exosomes^7,30^. Moreover, miRNAs can be found outside the cells, packaged in shedding vesicles, apoptotic bodies or forming complexes with RNA-binding proteins, such as Ago2, or lipid carriers as HDL (Apo-AI staining)^7,16,17^. Interestingly, we showed that both Ago2 and Apo-AI colocalize with NETs but not with each other, leading us to wonder whether the differential release of NET-miRs is a cause or consequence of distinct carriers (e.g., RNA-binding proteins versus lipid carriers, respectively). We also verified that the miRNA-142-3p carried by NETs could be transferred to macrophages upon cell interaction with NETs, leading to a reduced expression of PKCα. We found evidence that miRNA-142-3p can participate as a regulator of inflammatory mediators in macrophages exposed to NETs and LPS, controlling excessive TNF-α production, through PKCα down-modulation. Uncontrolled PKC activation and concomitant excess of pro-inflammatory cytokines can lead monocytes/macrophages to a desensitized phenotype, hindering subsequent immune responses^31^. Therefore, the presence of miRNAs in NET regulating inflammatory mediators could be interpreted as a negative feedback loop, regulating excessive responses and maintaining optimal tuning of the inflammatory reaction. In monocyte/macrophages, the PKC pathway is involved in processes of adhesion/migration, M1/M2 polarization, activation of TLRs and production of inflammatory cytokines. Accordingly, we can infer that the action of miRNA-142-3p may also affect those processes, implying in a wide level of regulation of cellular functions following interaction with NETs^24,32–36^.

The presence of miRNAs associated with the NET scaffold suggests that NETs may represent a new miRNA vehicle, capable to delivering gene-based mediators, thus favoring cell-to-cell communication, and regulating protein production in the miRNA recipient cell. Taking into account that many other miRNAs are also present in NET-enriched supernatants, future studies need to be performed to better comprehend and decipher the role of NETs in cell-to-cell communication and innate immune response regulation.

## Material and Methods

### Ethics statement

All experimental procedures involving human cells were performed with samples obtained after written informed consent, and all procedures and methods involving human biological samples were carried out in accordance with the guidelines and regulations approved by the Institutional Research Ethics Committee for Human Subjects of Hospital Clementino Fraga Filho (Universidade Federal do Rio de Janeiro, Brazil) protocol number: 4261 015400005257.

### Neutrophil purification

Human neutrophils from buffy coats of healthy blood donors were isolated by density gradient centrifugation (Sigma, USA) followed by hypotonic lysis of the erythrocytes. Purified neutrophils (95% of the cells) were resuspended in RPMI 1640 medium (Sigma, USA) and kept on ice until use, as described^37^.

### Parasites

*Leishmania amazonensis* (MHOM/BR/75/Josefa), 5-days stationary phase promastigotes grown on Schneider medium (Sigma, USA), with 10% heat inactivated fetal calf serum at 26 °C, were collected, washed in PBS, and fixed in 4% formaldehyde for 1 h. The parasites were extensively washed with PBS and used throughout.

### Preparation of the amyloid fibrils from A25T-transthyretin (TTR)

TTR was expressed using a heterologous system, and an A25T mutant was purified as described^3^. A25T was further purified using a polymyxin B-conjugated resin to ensure endotoxin removal as described by the manufacturers (Thermo Fisher, USA). A25T was aggregated by incubation in pH 7.3 at 37 °C with mild agitation for 2 weeks until amyloid fibrils formed.

### Neutrophil extracellular trap (NET) release assay

Neutrophils (8 × 10^6^) were incubated with PMA (100 ng/ml), fixed *Leishmania* promastigotes (5 parasites/1 neutrophil ratio), A25T (3 µM) or IL-8 (50 ng/mL, Biolegend, USA) for 3 h at 35 ^o^C with 5% CO_2_. The cells were centrifuged, and NET-DNA was quantified in the supernatants as previously described^37^ using the PicoGreen dsDNA kit (Invitrogen, USA) according to manufacturer’s instructions.

### LDH assay

Neutrophils were incubated with fixed *Leishmania* promastigotes (5 parasites/1 neutrophil ratio), 100 nM PMA or 50 ng/ml IL-8 for 3 h (37 °C, 5% CO_2_), and cell viability was determined according to the activity of lactate dehydrogenase (LDH) in the culture supernatants using a CytoTox® Kit (Promega, USA) according to the manufacturer’s instructions. Results, read at 490 nm, were expressed as percentages of released LDH compared to control cells lysed with 0.8% Triton X-100.

### Small RNA extraction

Small RNA was prepared from NET-enriched supernatants obtained from 12 different neutrophil donors using the miRNeasy Mini Kit (Qiagen, USA), which 6 donors were used for the PMA, La and A25T treatment and other 6 donors for PMA and IL-8 treatment. To allow for normalization of sample-to-sample variation in RNA isolation, cDNA synthesis, and real-time PCR, synthetic *C. elegans* miRNAs cel-miR-39, cel-miR-54, and cel-miR-238 (Applied Biosystems, USA) were added as a mixture of 25 pmol of each oligonucleotide in a 5 µl total volume to each denatured sample (i.e., after combining the sample with denaturing solution, Qiazol). The small RNA and miRNA concentration values were assessed through an Agilent 2100 RNA Bioanalyzer, using the small RNA kit (Agilent Technologies, USA).

### Microarray reanalysis

Global neutrophil miRNA expression normalized data available at the NCBI Gene Expression Omnibus (GEO) under accession GSE18999^15^ and at the EMBL-EBI ArrayExpress under accession E-TABM-1097^14^, were imported into the R statistical package version 2.922, and statistical analyses was performed for selecting miRNAs expressed by neutrophils regardless of the stimuli (Supplementary Table 1).

### Quantitative RT-PCR

Real time PCR was carried out using 20 ng of miRNA in 96-well plates. The Taqman miRNA assays (Thermo Fisher, USA) were prepared using a Taqman miRNA reverse transcription kit, Taqman Universal Master Mix II and no UNG according to the manufacturer’s instructions (Thermo Fisher, USA). The 96-well plates were read using a StepOne Plus Real-Time PCR system (Applied Biosystems, USA). The Taqman miRNA Assays ID numbers are listed in Supplementary Table 1. For the IL-8 assays we used 350 pg of miRNA as the initial material for each well. To avoid donor-to-donor variation, we performed a donor-paired analysis for all NET activators previously described, using PMA as a standard. The data were analyzed by fitting four-parameter sigmoid curves to the Rn data using the qPCR library^38^ for the R statistical package version 2.922. Spiked-in cell-miR-238-5p and cel-miR-54-5p, used for the normalization, were selected by the geNorm method^39^. The normalized data were used for heatmap construction and clustering using ClustVis^40^. We chose average as the clustering method and correlation as the clustering distance. The comparison of the means of the normalized gene expression values between the groups was performed by a nonparametric one-way ANOVA with 1,000 unrestricted permutations, followed by post-hoc pair-wise comparisons with a Bonferroni adjustment using a nonparametric t-test, also with 1,000 permutations^41^. The results were represented in graphs displaying the mean ± standard error of mean of the expression levels of the test groups (La and A25T), in relation to the control group (PMA). Two-tailed levels of significance less than or equal to 0.01 and 0.05 were considered as “highly significant” and “significant”, respectively.

### Live-cell imaging

Neutrophils (10^5^/well) were seeded in culture dishes equipped with glass bottoms (Corning, USA), in RPMI medium containing no phenol red with SYTOX Green (Molecular Probes, USA) at a 10 nM concentration and a TYE^TM^-665 labeled hsa-miR142-3p locked nucleic acid (LNA) detection probe (Exiqon, USA) at a 5 μM concentration. The cells were stimulated with 100 nM PMA, live *Leishmania* promastigotes (1 parasites/1 neutrophil ratio), A25T amyloid fibrils (3 µM) or IL-8 (50 ng/mL, Biolegend, USA) and photographed using a Leica DMI 6000 inverted microscope equipped with Leica Application Suite (LAS) software, a monochrome digital camera (DFC 360FX) and bandpass filters for excitation at 450-490 nm (long-pass emission at 515 nm) and 546–612 nm (long-pass emission at 590 nm). Images were taken after 120 minutes of incubation and frames were merged using Adobe Photoshop CS6 Software.

### Immunofluorescence

Neutrophils (10^5^/well) were seeded in culture dishes with poly-L-lysine-coated glass coverslips (Corning, USA), and incubated with 100 nM PMA or *Leishmania* promastigotes (1 parasite/1 neutrophil ratio) for 90 min at 35 ^o^C with 5% CO_2_ and fixed in 4% formaldehyde. Following, slides were stained with primary antibodies anti-Argonaute-2 and -Apoliprotein-AI (Abcam, USA), anti-elastase (Calbiochem, USA) or anti-DNA/histone H1 (Millipore, USA), followed by secondary antibodies goat-anti-rabbit or anti-mouse Alexa Fluor 488 or 546 (Molecular Probes, USA). The slides were mounted in ProLong Gold Antifade Mountant with DAPI (Thermo Fisher). Confocal images were taken in a Zeiss LSM 710.

### THP-1 cells

The human monocytic leukemia cell line THP-1 (ATCC: TIB202TM) was maintained in RPMI 1640 medium (LGC Bio, Brazil) supplemented with 10% heat-inactivated fetal calf serum (Cultilab, Brazil) and penicillin–streptomycin and differentiated into macrophages by treating them with 100 ng/mL of PMA for 2 days. Then, the cells were washed three times with PBS and incubated with fresh medium for an additional 3 days before starting the experiments. Macrophage differentiation was evaluated by flow cytometry for CD68 marker (BD Biosciences, USA) (Supplementary Fig. S5).

### Quantitative RT-PCR of NET treated THP-1 cells

Differentiated THP-1 macrophages were exposed for 2 h to PMA-induced NET, following washing and complete RNA extraction using miRNeasy Mini Kit (Qiagen, USA). The mature microRNA and microRNA primary transcripts were quantified in five independent experiments using a Taqman miRNA reverse transcription kit, Taqman Universal Master Mix II and no UNG and specific taqman probes for hsa-miR-142-3p (microRNA mature form) and hsa-pri-miR-142 (primary microRNA transcript) and RNU44 and GUSB as normalizing genes, respectively, all from Thermo Fisher (USA), according to the manufacturer’s instructions. The data were analyzed by fitting four-parameter sigmoid curves to the Rn data using the qPCR library^38^ for the R statistical package version 2.922. The comparison of the means of the normalized gene expression was performed by a nonparametric one-way ANOVA with 1,000 unrestricted permutations, followed by post-hoc pair-wise comparisons with a Bonferroni adjustment using a nonparametric t-test, also with 1,000 permutations^41^. The results were represented in graphs displaying the mean ± standard error of mean of the expression levels.

### PKCα and TNF-α analysis

THP-1 cells differentiated into macrophages in 24-well plates (Costar, USA) for PKC experiments, or in 96-well plates (Costar, USA) for TNF-α assays, were exposed to PMA-induced NET (6.5 µg/ml) associated or not with specific TYE™-665 labeled hsa-miR142-3p inhibitor probe (50 nM, Exiqon, USA) or TYE™-665 labeled scramble sequence control (50 nM, Exiqon, USA), or treated with cytochalasin D (10 µg/ml, Sigma, USA) before NET exposure. Salmon sperm DNA (6.5 µg/ml Thermo Fisher, USA) was used as a control. The specific miRNA inhibitor and the scramble control were pre-incubated (30 min) with NETs before cell exposure when necessary. After 2 h, cells were washed and complete culture medium was added. The expression of PKCα was evaluated by flow cytometry 18 h after treatment. For TNF-α assays, cells were submitted to the same treatments and also exposed to LPS (100 ng/ml; Sigma, USA), combined or not with NETs, miRNA inhibitor/scramble and a PKC inhibitor (Go6383, 50 nM; Tocris Bioscience, USA). Cell culture supernatants were collected 6 h later and TNF-α levels were quantified by ELISA (ImmunoTools, Germany).

### Flow cytometry

THP-1 were detached from culture dishes in ice-cold PBS using cell-scraper, and then incubated during 15 min in blocking solution (PBS plus 2 mM EDTA, 1% BSA, 25% human serum, and 25% mouse serum). Then, cells were permeabilized for 15 min (Intracellular Fixation & Permeabilization Buffer Set; Thermo Fisher, USA), and stained with mouse anti-CD68-Alexa 647 (BD Bioscience, USA) for evaluating cell differentiation (Supplementary Fig. S5), or rabbit anti-PKCα (Cell Signalling, USA), for 20 min in blocking solution. For PKCα staining, cells were washed and incubated with goat anti-rabbit-Alexa 488 (BD Bioscience, USA) for 20 min in blocking solution. Data were acquired with a BD Canto II flow cytometer using BD FACSDiva software (BD Bioscience, USA). Data were analyzed using FlowJo v10 (TreeStar Software, USA).

## Supplementary information

Supplementary Dataset 1.
