# Supplementary material for: Neutrophil extracellular trap-enriched supernatants carry microRNAs able to modulate TNF-α production by macrophages
Source: Sci Rep. 2020 Feb 17;10:2715. doi: 10.1038/s41598-020-59486-2 (PMC7026108; doi:10.1038/s41598-020-59486-2)
Supplement: Supplementary file 1 — Supplementary Dataset 1. [file 41598_2020_59486_MOESM1_ESM.docx]

**SUPPLEMENTARY INFORMATION FOR**

**Neutrophil extracellular trap-enriched supernatants carry microRNAs able to modulate TNF-α production by macrophages.**

Leandra Linhares-Lacerda^1^*^,#^, Jairo Ramos Temerozo^2,3,#^, Marcelo Ribeiro-Alves^4^, Estefania P. Azevedo^5^, Andres Mojoli^2^, Michelle T. C. Nascimento^1,5^, Gustavo Silva-Oliveira^1^, Wilson Savino^2,3^, Debora Foguel^5^, Dumith Chequer Bou-Habib^2,3^, Elvira M. Saraiva^1^*.

1 - Laboratory of Immunobiology of Leishmaniasis, Department of Immunology, Paulo de Goes Institute of Microbiology, Federal University of Rio de Janeiro, Rio de Janeiro, Brazil.

2 - Laboratory on Thymus Research, Oswaldo Cruz Institute, Oswaldo Cruz Foundation, Rio de Janeiro, Brazil.

3 - National Institute of Science and Technology on Neuroimmunomodulation, Oswaldo Cruz Institute, Oswaldo Cruz Foundation, Rio de Janeiro, Brazil.

4 - HIV/AIDS Clinical Research Center, Evandro Chagas National Institute of Infectology, Oswaldo Cruz Foundation, Rio de Janeiro, Brazil.

5 - Leopoldo de Meis Institute of Medical Biochemistry, Federal University of Rio de Janeiro, Rio de Janeiro, Brazil, Brazil.

# Equally contributed

***Corresponding authors:**

Elvira M. Saraiva [E-mail: [esaraiva@micro.ufrj.br](mailto:esaraiva@micro.ufrj.br)] and Leandra Linhares-Lacerda [[leandralacerda@gmail.com](mailto:leandralacerda@gmail.com)]

Universidade Federal do Rio de Janeiro

Av. Carlos Chagas Filho 373, Bloco D, sala D1-44

Ilha do Fundão, Rio de Janeiro, RJ

Brazil, 21941-902

Phone: (55-21) 3938.0363 Fax: (55-21) 2562-6789

**
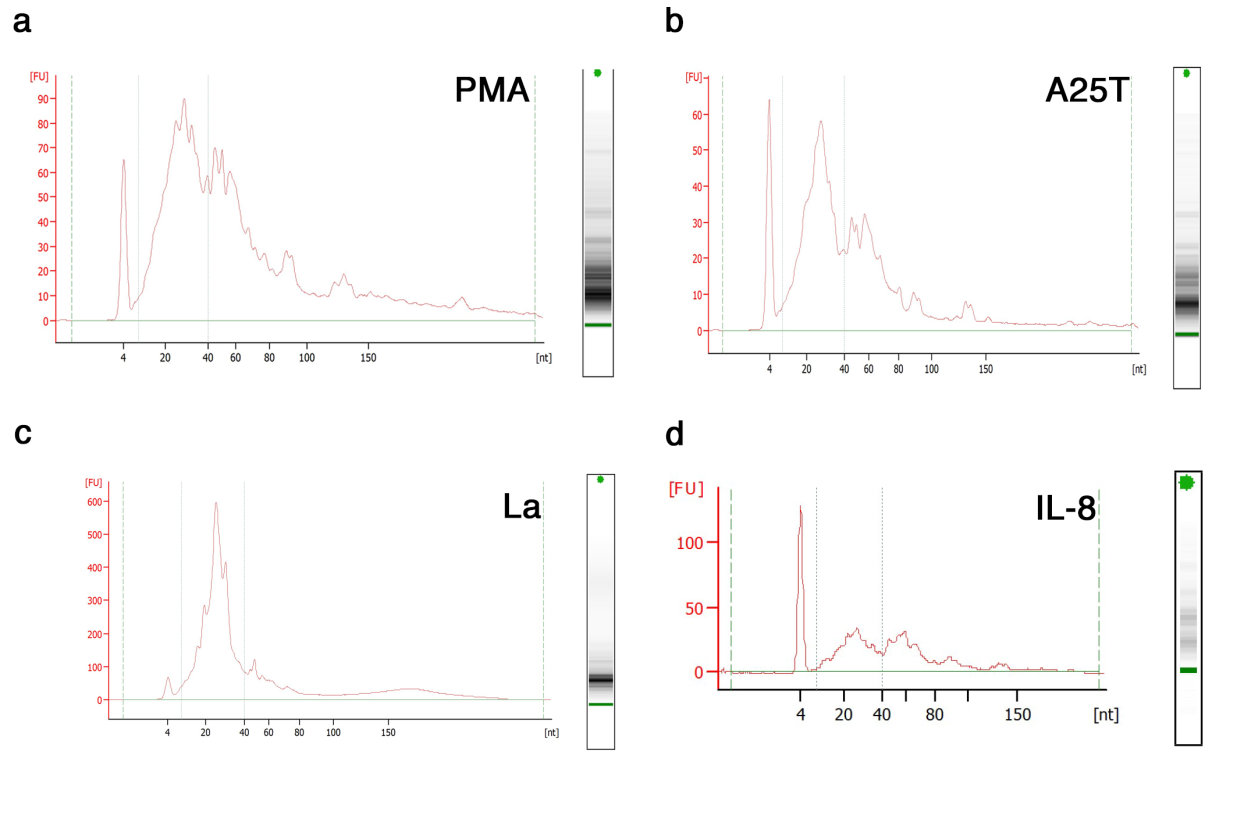
**

**Supplementary Figure S1: Bioanalyzer profiles from NET`s enriched supernatant from PMA, IL-8, A25T and La.** Representative profiles from 6 independent experiments. Abbreviations: PMA, phorbol myristate acetate; A25T, amyloid fibrils; IL-8, Interleukin-8; La, *Leishmania amazonensis.*

**Supplementary Figure S2: NET induction is not associated with lactate dehydrogenase (LDH) release.** (**A**) Neutrophils from healthy donors were exposed to three different activators, including *L. amazonensis* promastigotes (La; ratio of 5 parasites/neutrophil), PMA (100 nM) and IL-8 (50 ng/mL) for 3 h. **(A)** Detection of DNA (PicoGreen dsDNA kit) in supernatants as NET formation for each stimulus. (**B**) Measurement of lactate dehydrogenase (LDH) in supernatants for each stimulus. Results are from 3 independent experiments using one-way ANOVA, with Bonferroni post-hoc test, displaying mean ± standard error of mean of the test group. *p < 0.05.


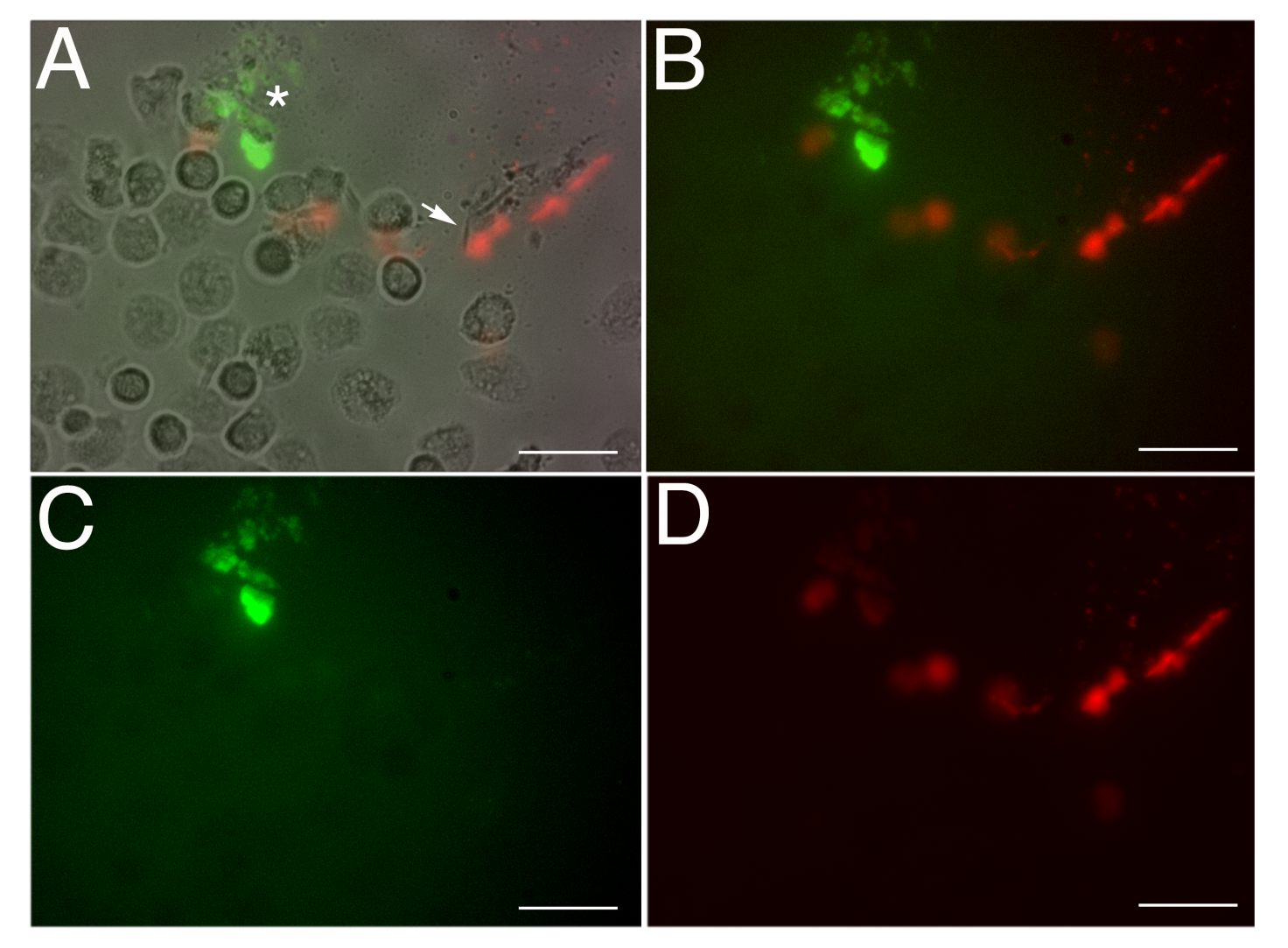


**Supplementary Figure S3:** **Live-cell imaging of NET formation in two different channels for up to 120 minutes.** SYTOX Orange at 10 nM was used for NET detection, and a FAM-labeled hsa-miR-181a-5p locked nucleic acid (LNA) detection probe at 5 μM for hsa-miR-181a-5p detection (depicted in green). The picture reveals NETs with the DNA scaffold forming a web-like structure containing trapped *Leishmania* promastigotes (arrow). The hsa-miR-181a-5p staining was only seen in dead neutrophils, most likely as an artifact due to the stick nature of dead cells (asterisk). The pictures show the merge of the two channels and DIC, with some viable neutrophils (**A**). We observed a weak DNA staining and hsa-miR-181a-5p (asterisk); however, in the stained NET the hsa-miR-181a-5p was not detected (**B**). (**C**) The picture shows the FAM-miR-181a-5p detection probe (green). (**D**) NET staining by SYTOX Orange. Bars: 25μm.

**
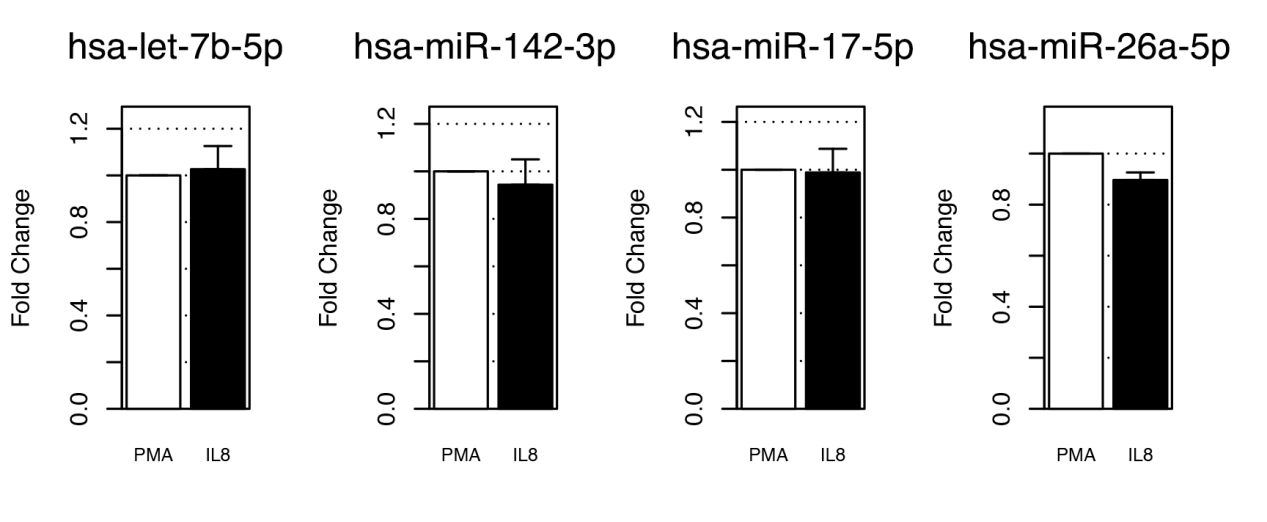
**

**Supplementary Figure S4: NET-associated miRNAs (NET-miRs) in IL-8-activated neutrophils**. Bar plot showing the miRNA expression between the activators (PMA and IL-8). Results were represented in graphs displaying the expression levels mean ± standard error of mean of the test group (IL-8) relative to the control group (PMA). To avoid donor to donor variation we performed donor-paired analysis. Abbreviations: PMA, phorbol myristate acetate; IL-8, Interleukin-8.

**Supplementary Figure S5: Expression of differentiation marker CD68 in PMA-differentiated THP-1 macrophages.** Representative histogram of CD68 expression in PMA-differentiated THP-1 macrophages 5 days after PMA treatment.

**Supplementary Tables:**

**Supplementary Table S1: miRNA significantly expressed by neutrophil regardless the stimuli from two microarray data reanalysis (see methods section for more information).**

|  | **miRNA from microarray** | **Mean** | **SEM⊗** |
| --- | --- | --- | --- |
| 1 | hsa-let-7a | 7.90 | 0.11 |
| 2 | hsa-let-7b | 7.90 | 0.50 |
| 3 | hsa-let-7b* | 6.08 | 1.33 |
| 4 | hsa-let-7c | 4.93 | 0.62 |
| 5 | hsa-let-7d | 6.16 | 0.23 |
| 6 | hsa-let-7d* | 6.89 | 1.25 |
| 7 | hsa-let-7f | 7.31 | 0.15 |
| 8 | hsa-let-7f-1* | 5.28 | 1.15 |
| 9 | hsa-let-7g | 6.96 | 0.12 |
| 10 | hsa-let-7g* | 4.42 | 1.01 |
| 11 | hsa-let-7i | 6.68 | 0.49 |
| 12 | hsa-miR-101 | 5.17 | 0.28 |
| 13 | hsa-miR-103 | 7.34 | 0.12 |
| 14 | hsa-miR-106b | 7.69 | 0.26 |
| 15 | hsa-miR-107 | 7.07 | 0.15 |
| 16 | hsa-miR-1180 | 6.33 | 1.43 |
| 17 | hsa-miR-1207-5p | 6.35 | 1.44 |
| 18 | hsa-miR-122 | 5.48 | 1.26 |
| 19 | hsa-miR-1224-3p | 6.17 | 1.35 |
| 20 | hsa-miR-1224-5p | 5.87 | 1.37 |
| 21 | hsa-miR-1225-3p | 4.87 | 0.86 |
| 22 | hsa-miR-1225-5p | 5.20 | 1.17 |
| 23 | hsa-miR-1227 | 4.45 | 0.97 |
| 24 | hsa-miR-1228* | 6.99 | 1.54 |
| 25 | hsa-miR-1229 | 4.61 | 1.04 |
| 26 | hsa-miR-1234 | 6.23 | 0.81 |
| 27 | hsa-miR-1236 | 4.76 | 1.04 |
| 28 | hsa-miR-1238 | 5.29 | 1.13 |
| 29 | hsa-miR-1260 | 8.39 | 0.94 |
| 30 | hsa-miR-1268 | 6.61 | 1.49 |
| 31 | hsa-miR-1274b | 7.43 | 1.20 |
| 32 | hsa-miR-1280 | 6.42 | 1.28 |
| 33 | hsa-miR-1281 | 7.56 | 1.28 |
| 34 | hsa-miR-1284 | 4.90 | 1.09 |
| 35 | hsa-miR-1296 | 6.06 | 1.33 |
| 36 | hsa-miR-1300 | 5.78 | 1.32 |
| 37 | hsa-miR-1306 | 6.55 | 1.46 |
| 38 | hsa-miR-1308 | 5.76 | 1.31 |
| 39 | hsa-miR-1322 | 5.10 | 1.19 |
| 40 | hsa-miR-136 | 4.81 | 1.07 |
| 41 | hsa-miR-140-3p | 5.63 | 0.12 |
| 42 | hsa-miR-140-5p | 6.60 | 0.18 |
| 43 | hsa-miR-142-3p | 9.68 | 0.72 |
| 44 | hsa-miR-142-5p | 7.09 | 0.16 |
| 45 | hsa-miR-1471 | 5.46 | 1.29 |
| 46 | hsa-miR-149 | 5.53 | 1.19 |
| 47 | hsa-miR-1538 | 5.34 | 1.20 |
| 48 | hsa-miR-1539 | 4.82 | 1.11 |
| 49 | hsa-miR-15a | 9.86 | 0.06 |
| 50 | hsa-miR-15a* | 5.19 | 1.20 |
| 51 | hsa-miR-15b | 9.02 | 0.06 |
| 52 | hsa-miR-16 | 10.42 | 0.04 |
| 53 | hsa-miR-16-2* | 4.68 | 1.07 |
| 54 | hsa-miR-17 | 5.42 | 0.39 |
| 55 | hsa-miR-181a | 5.31 | 0.54 |
| 56 | hsa-miR-181b | 5.65 | 1.30 |
| 57 | hsa-miR-181d | 5.84 | 1.36 |
| 58 | hsa-miR-1825 | 7.15 | 1.40 |
| 59 | hsa-miR-188-5p | 5.88 | 1.36 |
| 60 | hsa-miR-1909* | 6.68 | 1.47 |
| 61 | hsa-miR-1910 | 6.22 | 1.39 |
| 62 | hsa-miR-1915 | 5.94 | 0.93 |
| 63 | hsa-miR-194* | 4.90 | 1.15 |
| 64 | hsa-miR-195* | 5.42 | 1.22 |
| 65 | hsa-miR-197 | 8.41 | 0.74 |
| 66 | hsa-miR-19a | 5.64 | 0.24 |
| 67 | hsa-miR-19b | 7.38 | 0.18 |
| 68 | hsa-miR-19b-1* | 4.55 | 1.08 |
| 69 | hsa-miR-200c* | 4.92 | 1.13 |
| 70 | hsa-miR-206 | 6.62 | 1.46 |
| 71 | hsa-miR-20a | 7.00 | 0.27 |
| 72 | hsa-miR-21 | 8.91 | 0.19 |
| 73 | hsa-miR-214 | 4.87 | 1.16 |
| 74 | hsa-miR-22 | 5.96 | 0.16 |
| 75 | hsa-miR-221 | 5.48 | 1.01 |
| 76 | hsa-miR-223 | 13.80 | 0.17 |
| 77 | hsa-miR-23a | 8.97 | 0.24 |
| 78 | hsa-miR-24 | 7.70 | 0.15 |
| 79 | hsa-miR-25 | 6.50 | 0.32 |
| 80 | hsa-miR-26a | 7.48 | 0.26 |
| 81 | hsa-miR-26b | 8.07 | 0.30 |
| 82 | hsa-miR-27a | 8.83 | 0.08 |
| 83 | hsa-miR-27b* | 5.05 | 1.19 |
| 84 | hsa-miR-297 | 5.33 | 1.29 |
| 85 | hsa-miR-299-5p | 4.84 | 1.05 |
| 86 | hsa-miR-29a | 7.92 | 0.17 |
| 87 | hsa-miR-29b | 7.22 | 0.27 |
| 88 | hsa-miR-29b-1* | 4.62 | 1.11 |
| 89 | hsa-miR-29c | 6.79 | 0.22 |
| 90 | hsa-miR-300 | 4.98 | 1.11 |
| 91 | hsa-miR-30b | 4.96 | 0.58 |
| 92 | hsa-miR-30c | 5.90 | 0.63 |
| 93 | hsa-miR-32* | 7.60 | 1.10 |
| 94 | hsa-miR-320d | 4.53 | 0.72 |
| 95 | hsa-miR-324-3p | 6.04 | 1.31 |
| 96 | hsa-miR-328 | 7.22 | 1.43 |
| 97 | hsa-miR-331-3p | 4.60 | 0.44 |
| 98 | hsa-miR-335* | 4.85 | 1.07 |
| 99 | hsa-miR-338-3p | 7.13 | 0.29 |
| 100 | hsa-miR-338-5p | 5.65 | 1.30 |
| 101 | hsa-miR-346 | 4.96 | 1.14 |
| 102 | hsa-miR-34a | 5.00 | 1.19 |
| 103 | hsa-miR-34b | 6.11 | 1.02 |
| 104 | hsa-miR-382 | 5.12 | 1.24 |
| 105 | hsa-miR-423-5p | 4.97 | 1.11 |
| 106 | hsa-miR-424 | 5.40 | 0.28 |
| 107 | hsa-miR-425 | 5.44 | 0.31 |
| 108 | hsa-miR-432 | 5.94 | 1.37 |
| 109 | hsa-miR-432* | 5.00 | 1.11 |
| 110 | hsa-miR-483-3p | 7.45 | 1.25 |
| 111 | hsa-miR-485-3p | 6.54 | 1.43 |
| 112 | hsa-miR-520d-3p | 5.24 | 1.23 |
| 113 | hsa-miR-539 | 5.63 | 1.27 |
| 114 | hsa-miR-548d-5p | 4.90 | 1.13 |
| 115 | hsa-miR-550* | 4.86 | 1.08 |
| 116 | hsa-miR-574-3p | 8.03 | 0.91 |
| 117 | hsa-miR-574-5p | 7.87 | 1.04 |
| 118 | hsa-miR-595 | 6.87 | 1.51 |
| 119 | hsa-miR-605 | 5.31 | 1.18 |
| 120 | hsa-miR-609 | 5.38 | 1.18 |
| 121 | hsa-miR-610 | 5.50 | 1.27 |
| 122 | hsa-miR-615-3p | 5.84 | 1.28 |
| 123 | hsa-miR-623 | 5.81 | 1.34 |
| 124 | hsa-miR-629* | 5.23 | 1.15 |
| 125 | hsa-miR-631 | 6.04 | 1.33 |
| 126 | hsa-miR-634 | 3.95 | 0.83 |
| 127 | hsa-miR-636 | 4.95 | 1.16 |
| 128 | hsa-miR-638 | 6.49 | 0.63 |
| 129 | hsa-miR-640 | 5.20 | 1.17 |
| 130 | hsa-miR-647 | 5.76 | 1.23 |
| 131 | hsa-miR-654-3p | 6.01 | 1.30 |
| 132 | hsa-miR-664 | 5.51 | 1.06 |
| 133 | hsa-miR-675* | 4.92 | 1.09 |
| 134 | hsa-miR-720 | 12.23 | 0.04 |
| 135 | hsa-miR-765 | 5.56 | 1.27 |
| 136 | hsa-miR-766 | 7.10 | 1.40 |
| 137 | hsa-miR-877* | 6.66 | 1.45 |
| 138 | hsa-miR-885-3p | 4.97 | 1.34 |
| 139 | hsa-miR-885-5p | 7.01 | 1.36 |
| 140 | hsa-miR-923_v12.0 | 6.69 | 0.16 |
| 141 | hsa-miR-92a | 4.99 | 0.46 |
| 142 | hsa-miR-93 | 5.89 | 0.42 |
| 143 | hsa-miR-933 | 3.45 | 0.84 |
| 144 | hsa-miR-937 | 5.93 | 1.30 |
| 145 | hsa-miR-939 | 5.88 | 1.36 |
| 146 | hsa-miR-940 | 6.03 | 0.60 |

⊗SEM= standard error from mean

**Supplementary Table S2: miRNA expression levels of the groups from quantitative PCR analysis.**

| Assay_ID* | microRNA name | Global.p.value | log2(LA/PMA)# | p.value | log2(A25T/PMA)# | p.value | log2(A25T/La)# | p.value |
| --- | --- | --- | --- | --- | --- | --- | --- | --- |
| 002619 | hsa-let-7b-5p | 0.09 | -0.27 | 0.06 | -0.09 | 1.00 | 0.18 | 0.65 |
| 002283 | hsa-let-7d-5p | 0.69 | 0.16 | 1.00 | 0.03 | 1.00 | -0.12 | 1.00 |
| 002282 | hsa-let-7g-5p | 0.53 | -0.03 | 1.00 | 0.09 | 1.00 | 0.12 | 0.95 |
| 002221 | hsa-let-7i-5p | 0.08 | -0.38 | 0.14 | 0.01 | 1.00 | 0.39 | 0.18 |
| 000439 | hsa-miR-103a-3p | 0.98 | -0.02 | 1.00 | -0.06 | 1.00 | -0.05 | 1.00 |
| 000442 | hsa-miR-106b-5p | 0.55 | 0.30 | 0.64 | 0.18 | 1.00 | -0.12 | 1.00 |
| 002847 | hsa-miR-1180-3p | 0.42 | -0.03 | 1.00 | -0.11 | 0.61 | -0.08 | 1.00 |
| 002766 | hsa-miR-1225-3p | 0.22 | 0.23 | 0.62 | -0.09 | 1.00 | -0.32 | 0.25 |
| 002764 | hsa-miR-1225-5p | 0.00 | 1.60 | 0.00 | -0.07 | 1.00 | -1.67 | 0.00 |
| 242525_mat | hsa-miR-1260b | 0.44 | 0.14 | 0.62 | 0.01 | 1.00 | -0.14 | 0.73 |
| 241042_mat | hsa-miR-1281 | 0.34 | 0.04 | 1.00 | -0.12 | 0.94 | -0.16 | 0.40 |
| 002234 | hsa-miR-140-3p | 0.57 | -0.11 | 0.94 | -0.01 | 1.00 | 0.10 | 1.00 |
| 001187 | hsa-miR-140-5p | 0.00 | 0.11 | 0.28 | 0.20 | 0.00 | 0.08 | 0.79 |
| 000464 | hsa-miR-142-3p | 0.00 | -0.31 | 0.03 | 0.05 | 1.00 | 0.36 | 0.00 |
| 000390 | hsa-miR-15b-5p | 0.11 | -0.22 | 0.30 | 0.05 | 1.00 | 0.26 | 0.13 |
| 000391 | hsa-miR-16-5p | 0.04 | -0.19 | 0.10 | 0.00 | 1.00 | 0.19 | 0.08 |
| 002308 | hsa-miR-17-5p | 0.10 | 0.03 | 1.00 | 0.20 | 0.14 | 0.17 | 0.28 |
| 000480 | hsa-miR-181a-5p | 0.44 | -0.15 | 0.60 | -0.10 | 1.00 | 0.05 | 1.00 |
| 002320 | hsa-miR-188-5p | 0.33 | -0.03 | 1.00 | -0.14 | 0.50 | -0.11 | 0.82 |
| 000497 | hsa-miR-197-3p | 0.10 | -0.33 | 0.12 | -0.09 | 1.00 | 0.24 | 0.45 |
| 000396 | hsa-miR-19b-3p | 0.03 | -0.17 | 0.10 | 0.03 | 1.00 | 0.19 | 0.03 |
| 000580 | hsa-miR-20a-5p | 0.00 | -0.05 | 1.00 | 0.22 | 0.02 | 0.27 | 0.01 |
| 000397 | hsa-miR-21-5p | 0.54 | 0.15 | 0.82 | -0.01 | 1.00 | -0.16 | 0.78 |
| 000524 | hsa-miR-221-3p | 0.87 | 0.00 | 1.00 | 0.03 | 1.00 | 0.03 | 1.00 |
| 002295 | hsa-miR-223-3p | 0.00 | -0.38 | 0.02 | 0.03 | 1.00 | 0.42 | 0.00 |
| 000399 | hsa-miR-23a-3p | NA | NA | 1.00 | NA | 1.00 | NA | 1.00 |
| 000402 | hsa-miR-24-3p | 0.16 | -0.14 | 0.62 | 0.06 | 1.00 | 0.21 | 0.17 |
| 000403 | hsa-miR-25-3p | 0.01 | -0.41 | 0.03 | -0.04 | 1.00 | 0.36 | 0.04 |
| 000405 | hsa-miR-26a-5p | 0.06 | -0.21 | 0.42 | 0.11 | 1.00 | 0.31 | 0.06 |
| 000407 | hsa-miR-26b-5p | 0.04 | -0.18 | 0.49 | 0.12 | 1.00 | 0.30 | 0.04 |
| 000408 | hsa-miR-27a-3p | 0.00 | -0.88 | 0.00 | -0.33 | 0.55 | 0.55 | 0.02 |
| 002112 | hsa-miR-29a-3p | 0.33 | -0.36 | 0.38 | -0.11 | 1.00 | 0.25 | 0.81 |
| 000419 | hsa-miR-30c-5p | 0.05 | -0.25 | 0.14 | 0.03 | 1.00 | 0.27 | 0.07 |
| 002161 | hsa-miR-324-3p | 0.80 | 0.08 | 1.00 | -0.08 | 1.00 | -0.16 | 0.90 |
| 000543 | hsa-miR-328-3p | 0.47 | -0.18 | 0.58 | -0.09 | 1.00 | 0.09 | 1.00 |
| 002658 | hsa-miR-338-5p | NA | NA | 1.00 | NA | 1.00 | NA | 1.00 |
| 002102 | hsa-miR-34b-3p | NA | NA | 1.00 | NA | 1.00 | NA | 1.00 |
| 002340 | hsa-miR-423-5p | 0.32 | -0.21 | 0.33 | -0.12 | 1.00 | 0.09 | 1.00 |
| 001026 | hsa-miR-432-5p | 0.25 | 0.17 | 0.76 | NA | 1.00 | NA | 1.00 |
| 002349 | hsa-miR-574-3p | 0.57 | -0.15 | 0.87 | -0.04 | 1.00 | 0.12 | 1.00 |
| 001575 | hsa-miR-610 | NA | NA | 1.00 | NA | 1.00 | NA | 1.00 |
| 002088 | hsa-miR-636 | 0.01 | 0.32 | 0.02 | 0.01 | 1.00 | -0.31 | 0.01 |
| 001582 | hsa-miR-638 | 0.45 | 0.16 | 1.00 | -0.14 | 1.00 | -0.29 | 0.58 |
| 002372 | hsa-miR-885-3p | NA | NA | 1.00 | NA | 1.00 | NA | 1.00 |
| 001090 | hsa-miR-93-5p | 0.79 | 0.03 | 1.00 | 0.06 | 1.00 | 0.03 | 1.00 |
| 002182 | hsa-miR-939-5p | 0.30 | -0.39 | 0.82 | -0.52 | 0.46 | -0.13 | 1.00 |

*Assay_ID from Thermo Fisher microRNA Taqman® Gene Expression Assays. The comparison of means of normalized gene expression values between groups were performed by a nonparametric one-way ANOVA with 1,000 unrestricted permutations (Global p-value), followed by post-hoc pair-wise comparisons with Bonferroni adjustment by a nonparametric t-test also with 1,000 permutations (p-value). #Results are represented by log Fold Change (base 2).
